# Rifapentine- and moxifloxacin-containing short-course regimens for mild spinal tuberculosis: study protocol for a multicenter, randomized, non-inferiority phase II clinical trial

**DOI:** 10.3389/fphar.2025.1684771

**Published:** 2025-12-19

**Authors:** Yue-E Wu, Jing Cao, Li Wang, Ying-Ji Wei, Hui-Xin Liu, Lei Qi, Wen Zhang, Feng Liu, Zhong-Jiao Zhu, Xu-Tao Fan, Sheng Wang, Cong Chen, Xiang-Ze Liu, Ying Li, Zhong-Zheng Sun, Jing-Bo Pan, Chao-Qun Yang, John Van Den Anker, Qiang Zhang, Wei Zhao

**Affiliations:** 1 Department of Clinical Pharmacy, Institute of Clinical Pharmacology, Key Laboratory of Chemical Biology (Ministry of Education), NMPA Key Laboratory for Clinical Research and Evaluation of Innovative Drug, School of Pharmaceutical Sciences, Qilu Hospital, Cheeloo College of Medicine, Shandong University, Jinan, China; 2 Department of Orthopaedic Surgery, Shandong Public Health Clinical Center, Shandong University, Jinan, China; 3 Department of Orthopedics, Linyi Mental Health Center, Linyi, China; 4 Department of Orthopaedic Surgery, Qilu Hospital of Shandong University, Jinan, China; 5 Department of Spine Surgery, Shandong Provincial Hospital Affiliated to Shandong First Medical University, Jinan, China; 6 Department of Orthopedic Surgery, Taian City Central Hospital, Taian, China; 7 Department of Spinal Surgery, Tengzhou Central People’s Hospital, Tengzhou, China; 8 Department of Spine Surgery, Affiliated Hospital of Jining Medical University, Jining, China; 9 Department of Spinal Surgery, Affiliated Hospital of Shandong Second Medical University, Weifang, China; 10 Department of Orthopedics, Weihai Municipal Hospital, Cheeloo College of Medicine, Shandong University, Weihai, China; 11 Department of Orthopedics, Weifang NO.2 People’s Hospital, Weifang, China; 12 Department of Tuberculosis, Zibo Infectious Diseases Hospital, Zibo, China; 13 Department of Spinal Surgery, PKUCare Luzhong Hospital, Zibo, China; 14 Department of Orthopedics, Yantaishan Hospital Affiliated to Binzhou Medical University, Yantai, China; 15 Department of Spine Surgery, Jinan Third People’s Hospital, Jinan, China

**Keywords:** mild spinal tuberculosis, short-course regimen, rifapentine, moxifloxacin, non-inferiority

## Abstract

**Background:**

Spinal tuberculosis is the most common form of osteoarticular infection, with recommended anti-tuberculosis treatment durations typically being long (9–18 months), even for mild cases, increasing the risks of drug resistance, toxicity, and poor patient adherence. This study aims to explore, in patients with mild spinal tuberculosis, whether a new combination of anti-tuberculosis drugs (containing rifapentine and moxifloxacin) could shorten the treatment duration.

**Methods and Analysis:**

This trial is an open-label, randomized, controlled, non-inferiority trial comparing the efficacy and safety of a short-course regimen [6-month anti-TB regimen] containing rifapentine and moxifloxacin with empirical long-course regimen [12-month anti-TB regimen] in the treatment of patients with mild spinal tuberculosis. Patients diagnosed with mild spinal tuberculosis who meet the inclusion and exclusion criteria will be recruited and randomized in a 1:1 ratio to either of the two arms. Empirical long-course regimen includes rifampin, isoniazid, pyrazinamide and ethambutol (2RHZE/10RH), while short-course regimen includes rifapentine, moxifloxacin, isoniazid and pyrazinamide (2HRtZM/4HRtM). The primary outcomes are TB-recurrence rate at 24 months after completion of treatment and proportion of participants with grade 3 or higher adverse events during treatment with study medications.

**Discussion:**

This trial will provide evidence whether a short-course regimen of anti-TB drugs (2HRtZM/4HRtM) is non-inferior to the empirical long-course regimen (2RHZE/10RH) in patients with mild spinal tuberculosis. If non-inferiority is established, it will contribute to a more optimized treatment for spinal tuberculosis.

**Clinical Trial Registration:**

This study is registered with https://clinicaltrials.gov/ct2/show/NCT06917495 as NCT06917495.

## 1 Introduction

Spinal tuberculosis is the most common type of osteoarticular tuberculosis, accounting for approximately 1%–3% of all tuberculosis cases and representing one of the most prevalent types of extrapulmonary tuberculosis (Meier, 1994; Li et al., 2023; WHO, 2024). As a country ranking third among the 30 countries with the highest tuberculosis burden globally, China faces considerable challenges from spinal tuberculosis, with a large number of new cases reported annually—its tuberculosis incidence reaching 52 per 100,000 population in 2023 (Li et al., 2023; WHO, 2023; WHO, 2024). Clinically, spinal tuberculosis poses severe health risks: it accounts for 62% of deaths associated with osteoarticular tuberculosis (Li et al., 2023), and post-tuberculosis sequelae such as spinal deformities, neurological disabilities, and paraplegia are common, significantly impairing patient’s quality of life and imposing long-term physical and psychological burdens (Igbokwe et al., 2023). For children and adolescents, these sequelae can lead to lifelong functional limitations, further exacerbating the public health impact (Igbokwe et al., 2023). Mild spinal tuberculosis, characterized by early-stage or localized spinal involvement (Oguz et al., 2008), constitutes a substantial proportion of spinal tuberculosis cases; these patients are primarily managed with drug therapy, which presents a unique opportunity to explore more optimized treatment strategies and to prevent further disease progression (Oguz et al., 2008).

Anti-tuberculosis (anti-TB) drug therapy is a crucial approach for treating spinal tuberculosis infections, especially in cases of mild spinal tuberculosis (Rajasekaran and Khandelwal, 2013). In guidelines, an extended treatment duration of 9–18 months for osteoarticular tuberculosis is recommended, which is 1.5–3 times longer than the standard 6-month regimen (2HRZE/4HR) typically used for extrapulmonary tuberculosis (Hongqi et al., 2022; WHO, 2022). Beyond the direct health hazards of the diease itself, this prolonged treatment brings additional societal and economic burdens: it increases the risk of drug toxicity (such as hepatotoxicity) (Yew et al., 2018; Maiwall et al., 2024), drug resistance (Zumla et al., 2014) — a growing concern globally as multidrug-resistant TB continues to thwart control efforts (Romano et al., 2020) — and poor patient adherence (Guix-Comellas et al., 2015). These issues not only reduce treatment effectiveness but also lead to increased medical costs and resource comsumption; for patient in low- and middle-income regions or with limited medical access, the long treatment cycle may result in treatment interruption, further worsening the diease burden (Tochukwu et al., 2023).

A pilot study initially explored the efficacy of 6-month and 12-month anti-TB treatments for spinal tuberculosis, showing similar clinical outcomes in both groups (Nene et al., 2019). In addition, a large multicenter randomized controlled trial demonstrated that a new 4-month anti-TB regimen containing isoniazid, rifapentine, moxifloxacin, and pyrazinamide was non-inferior to the standard 6-month regimen (2HRZE/4HR) in treating drug-sensitive pulmonary tuberculosis in terms of efficacy, safety, and tolerability—confirming the synergistic bactericidal potential of these four drugs (Dorman et al., 2021). These findings, combined with the unique pharmacological advantages of rifarentine and moxifloxacin in targeting spinal tuberculosis, provide critical insights into optimizing treatment for mild spinal tuberculosis.

Notably, the 2HRtZM/4HRtM regimen is not the only option but may be the most appropriate, balancing efficacy, safety, and clinical applicability. Alternatives containing bedaquiline or delamanid were excluded because they are primarily recommended for drug-resistant tuberculosis (WHO, 2022) with no evidence in drug-sensitive mild spinal tuberculosis, and their higher costs and stricter safety monitoring (e.g., cardiac QT interval monitoring for bedaquiline) limit accessibility in diverse medical settings—relevant given this study’s 14 centers across China with varying resources. In contrast, rifapentine and moxifloxacin are included in China’s National Drug Catalogue for Basic Medical Insurance (National Healthcare Security Administration, 2024), ensuring cost-effectiveness and broad accessibility.

Therefore, this study aims to investigate whether the rifapentine- and moxifloxacin-containing 6-month anti-TB regimen is non-inferior to a 12-month empirical regimen for the treatment of mild spinal tuberculosis in terms of efficacy and safety through a multicenter, randomized, phase II clinical trial—with the ultimate goal of reducing sequelae, alleviating disease burden, and improving accessibility of effective treatment, particularly in regions with varying levels of medical resources.

## 2 Methods and analysis

### 2.1 Study design

**FIGURE 1:**
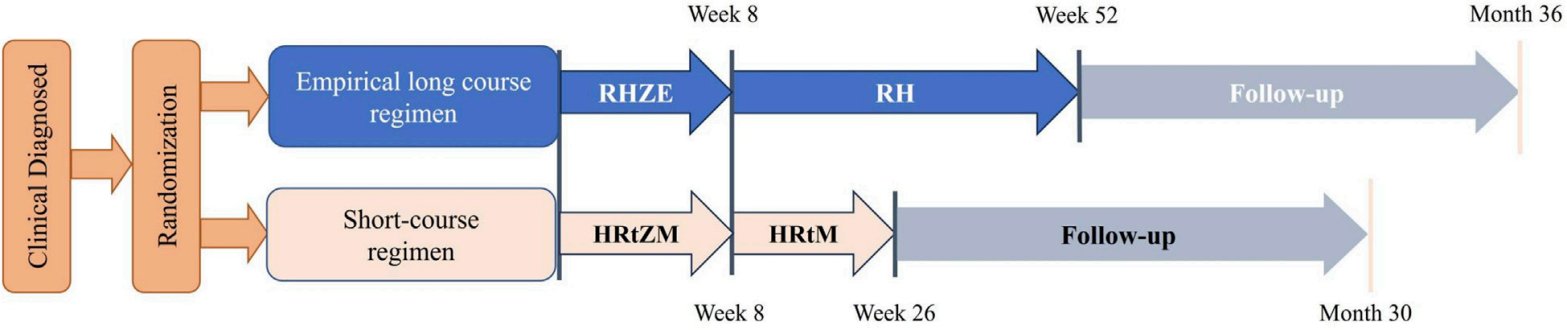
The flowchart of this trial.

This is an open-label, multicenter, non-inferiority randomized controlled trial designed to compare the efficacy and safety of a short-course regimen [6-month anti-TB regimen] containing rifapentine and moxifloxacin with an empirical long-course regimen [12-month anti-TB regimen] in the treatment of patients with mild spinal tuberculosis (Table 1). A total of 300 participants will be recruited from 14 study sites in China. The flowchart of this trial is shown in Figure 1.

**TABLE 1:** Diagnostic criteria of mild spinal tuberculosis.

Diagnostic criteria
➢ Mild to moderate neurologic deficits: American spinal injury association grade D or E
➢ Single vertebral involvement with central lesions or multiple vertebral involvement (<3) with marginal lesions
➢ Vertebral appendage tuberculosis without canal involvement
➢ Vertebral body collapse less than 1/3 of the total height
➢ The extent of paravertebral abscess is limited (Confined to single vertebral level, no retropharyngeal abscess or lumbar muscle abscess)
➢ No significant spinal kyphosis (Cobb angle <30°)
➢ No apparent spinal instability (Lateral X-ray shows thoracic sagittal displacement >2.5 mm, and lumbar sagittal displacement >4.5 mm or 15% of vertebral anterior-posterior diameter on static lateral X-ray are considered potentially unstable)

### 2.2 Population

The recruitment of eligible patients will be overseen by the clinicians from the attending research teams. Participants or their legal guardians, will be fully informed about the study, and written informed consent will be obtained from all participants or their guardians. Patients are eligible for recruitment if they meet all the inclusion criteria and do not meet any of the exclusion criteria (Table 2).

**TABLE 2:** The eligibility criteria.

Inclusion criteria	Exclusion criteria
Pre-randomization	
1. Age ≥12 years 2. Based on medical history, clinical manifestations, radiological exams, laboratory tests, and possible histological samples, the patient is diagnosed with mild spinal tuberculosis according to the criteria (Table 1) 3. Laboratory test values are obtained within 14 days prior to screening [①serum or plasma ALT ≤3 × ULN; ②serum or plasma TBIL ≤2.5 × ULN; ③serum or plasma creatinine level ≤2 × ULN; ④serum or plasma potassium level greater than or equal to 3.5 mmol/L (Max 5.5 mmol/L); ⑤hemoglobin level of 7.0 g/dL or higher (Max 17.2 g/dL for male, 15.1 g/dL for female); ⑥platelet count of 100,000/mm^3^ or higher (Max 300,000/mm^3^)] 4. Women of childbearing potential who are not surgically sterilized must agree to practice a barrier method of contraception or abstain from heterosexual intercourse during study drug treatment 5. Women of childbearing potential must have a negative pregnancy test at or within 7 days prior to screening 6. KPS ≥60 7. A verifiable address or residence location that is readily accessible for visiting, and willingness to inform the study team of any change of address during the treatment and follow-up period 8. Written informed consent	1. Pregnant or breastfeeding 2. Unable to take oral medications 3. Previously enrolled in similar studies 4. Spinal tumors or metastatic tumors 5. Patients with mental disorders or cognitive dysfunction 6. Have received any investigational drug in the past 3 months 7. Have received more than 5 days of treatment directed against active tuberculosis within 6 months preceding initiation of study drugs 8. Have received more than 5 days of systemic treatment with any one or more of the following drugs within 30 days preceding initiation of study drugs: Isoniazid, rifampin, rifambutin, rifapentine, ethambutol, pyrazinamide, kanamycin, amikacin, streptomycin, capreomycin, moxifloxacin, levofloxacin, gatifloxacin, ofloxacin, ciprofloxacin, other fluoroquinolones, ethionamide, prothionamide, cycloserine, terizidone, para-aminosalicylic acid, linezolid, clofazimine, delamanid or bedaquiline 9. Known history of prolonged QT syndrome 10. Weight less than 40.0 kg 11. Known allergy or intolerance to any of the study medications 12. Individuals will be excluded from enrollment if, at the time of enrollment, their Mtb isolate is already known to be resistant to any one or more of the following: rifampin, isoniazid, pyrazinamide, ethambutol, or fluoroquinolones 13. Other medical conditions that, in the investigator’s judgment, make study participation not be in the individual’s best interest
Post-randomization	
	14. No Mtb is identified in the screening, baseline, and week 2 samples 15. Mtb isolated from or tested by molecular assays (Xpert MTB/RIF) in samples obtained before or after the study are determined to be resistant to isoniazid, rifampin, or fluoroquinolones

ALT: alanine aminotransferase; KPS: karnofsky performance status; Mtb: *Mycobacterium tuberculosis*; TBIL: total bilirubin; ULN: upper limit of normal.

### 2.3 Randomization

A blocked randomization approach with stratification will be employed to allocate eligible patients into the short-course regimen [6-month anti-TB regimen] group and the empirical long-course regimen [12-month anti-TB regimen] group in a 1:1 ratio. The participants will be stratified according to the study sites. All research staff will undergo uniform training to ensure consistency across all study sites. The randomization list will be generated using SAS v9.4, and the allocation sequence will be carried out by the trial statistician via the central phone system. Both participants and investigators will be aware of the allocation, as the study will not be blinded.

### 2.4 Interventions

Participants will be randomly assigned to receive anti-TB drugs in either the short-course regimen or the empirical long-course regimen. Participants in the short-course regimen group will receive 8 weeks of daily treatment with isoniazid, rifapentine, pyrazinamide and moxifloxacin, followed by 18 weeks of daily treatment with isoniazid, rifapentine, and moxifloxacin (2HRtZM/4HRtM). Participants in the empirical long-course regimen group will receive 8 weeks of daily treatment with rifampin, isoniazid, pyrazinamide, and ethambutol, followed by 44 weeks of daily treatment with rifampin and isoniazid (2RHZE/10RH). All drugs will be administered orally once daily. The dosing regimen details are provided in Table 3.

**TABLE 3:** Dosing regimens.

Group	Medicine	Dosage		Durations
Short-course regimen (2HRtZM/4HRtM)	Rifapentine	≤41.2 kg	750 mg	26 weeks
		>41.3–48.7 kg	900 mg	
		>48.8–56.2 kg	1,050 mg	
		≥56.3 kg	1,200 mg	
	Moxifloxacin	400 mg		26 weeks
	Isoniazid	300 mg		26 weeks
	Pyrazinamide	<55 kg	1,000 mg	8 weeks
		≥55–75 kg	1,500 mg	
		>75 kg	2000 mg	
Empirical long course regimen (2RHZE/10RH)	Rifampin	600 mg		52 weeks
	Isoniazid	300 mg		52 weeks
	Pyrazinamide	<55 kg	1,000 mg	8 weeks
		≥55–75 kg	1,500 mg	
		>75 kg	2000 mg	
	Ethambutol	<55 kg	800 mg	8 weeks
		≥55–75 kg	1,200 mg	
		>75 kg	1,600 mg	

All drugs are administered orally, once a day.

### 2.5 Data collection

Participants will undergo clinical and laboratory assessments at predetermined timepoints (Table 4). In addition, to evaluate the exposure levels of anti-TB drugs in the body of participants, pharmacokinetic samples, including but not limited to blood, will be collected by using an opportunistic sampling method (Leroux et al., 2015). The samples will be collected at any time after the 14th dose of the anti-TB drugs, but no later than Week 8 after the start of treatment. Collection is recommended at weeks 2, 4, and 8. A total of 2-4 blood samples will be collected from each participant during the entire treatment period. The study follow-up will continue for 24 months after the completion of anti-TB treatment.

**TABLE 4:** The schedule of study assessments for this trial.

Visit	Baseline period	Treatment period ( ± 3 days)							Follow-up period ( ± 7 days)						Possible poor treatment response	Post early termination visit
Timing	-7days - 0 d	WK 2	WK 4	WK 8	WK 13	WK 17	WK 22	WK 26	MO 9	MO 12	MO 18	MO 24	MO 30	MO 36		
Informed consent	X															
Inclusion/Exclusion	X															
Randomization	X															
Demographics	X															
Contact information	X	X	X	X	X	X	X	X	X	X	X	X	X	X	X	X
Medical history and diagnosis	X															
Symptoms		X	X	X	X	X	X	X	X	X	X	X	X	X	X	X
Administration		X	X	X	X	X	X	X								
Concomitant medications		X	X	X	X	X	X	X	X	X	X	X	X	X	X	X
Recent medical record									X	X	X	X	X	X	X	
Height	X															
Weight	X	X	X	X	X	X	X	X	X	X						
Diabetes screening	X															
Visual tests			X													
Pregnancy testing	X															
Rapid molecular test	X															
Bacterial smear and culture	X	X	X	X	X	X	X	X	X	X	X	X	X	X	X	
Chest radiograph	X							X		X						
Neurological assessment	X	X	X	X	X	X	X	X	X	X	X	X	X	X	X	
Biochemical test	X	X	X	X	X	X	X	X	X	X						X
Blood tests	X	X	X	X	X	X	X	X	X	X						X
PK sampling		X	X	X												
Adverse events		X	X	X	X	X	X	X	X	X	X	X	X	X	X	X
CRF	X	X	X	X	X	X	X	X	X	X	X	X	X	X	X	X

X Fixed. PK, pharmacokinetics; CRF, case report form.

### 2.6 Outcomes

#### 2.6.1 Primary outcomes

The primary outcomes are TB-recurrence rate of spinal tuberculosis at 24 months after completion of treatment (efficacy outcome, non-inferiority aspect) and the proportion of participants with grade 3 or higher adverse events (AEs) during study medication (safety outcome, superiority aspect). The recurrence of spinal tuberculosis is defined by the reappearance of pain, with or without sinus formation, loosening or displacement of internal fixation on X-ray, and confirmed by postoperative computed tomography or magnetic resonance imaging showing increased local abscess, bone graft absorption, new sequestrum formation, or aggravated bone destruction (Ren et al., 2016). AEs are graded per CTCAE v5.0, with grade 3+ events defined as severe functional impairment, life-threatening complications, or death; causality is adjudicated by the Safety Monitoring Committee using WHO-UMC criteria.

#### 2.6.2 Secondary outcomes

The secondary outcomes are clinical cure at the end of therapy, TB-recurrence rate of spinal tuberculosis 12 months after completion of treatment, the proportion of participants who are culture negative at 8 weeks, the proportion of discontinuation of assigned treatment for a reason other than microbiological ineligibility, the incidence of AEs, the proportion of participants who have residual neurological dysfunction, and pharmacokinetic (PK) parameters and pharmacodynamic (PD) target attainment rate of the anti-TB drugs (Asin-Prieto et al., 2015; Verbeeck et al., 2016; Zuur et al., 2018). The determination of clinical cure needs to be based on a composite of clinical, laboratory (normal complete blood count and erythrocyte sedimentation rate), and radiological factors. Clinically, patients are considered “healed” at the end of therapy if they show marked improvement in spinal symptoms, including pain, tenderness, and paraspinal muscle spasm, along with a return to their pre-disease functional status, weight gain, and absence of residual instability or neurological deficits. Radiologically, healing is defined as substantial regression of epidural or paraspinal abscess/granulation tissue, marrow reconversion, and fatty reconstitution of the affected bone at the final follow-up (Nene et al., 2019). For PK parameters and PD target attainment rate, plasma concentrations of rifapentine and moxifloxacin are measured via validated liquid chromatography-tandem mass spectrometry (LC-MS/MS) from 2-4 blood samples collected per participant (Weeks 2, 4, 8). PK parameters (area under the concentration-time curve over 24 h [AUC_0–24h_]; maximum plasma concentration [C_max_]) are calculated using the NONMEM 7.4 software. PD targets (rifapentine: AUC_0–24h_/minimum inhibitory concentration [MIC] (Verbeeck et al., 2016; Zuur et al., 2018); moxifloxacin: AUC_0-24h_/MIC (Asin-Prieto et al., 2015) are based on anti-TB pharmacology; the attainment rate is the proportion of participants meeting both targets.

### 2.7 Sample size

We estimated the sample size based on the non-inferiority hypothesis that the TB-recurrence rate of spinal tuberculosis at 24 months after completion of treatment of the short-course regimen will be no worse than the empirical long-course regimen. It is assumed that the TB-recurrence rate in the empirical long-course group is 5% and the rate will be the same in the short-course regimen arm. Considering a 10% dropout rate, a sample size of 150 per arm (300 in total) will give 80% power (one-sided type I error of 5%) to demonstrate a 6.6% non-inferiority margin (Dorman et al., 2021) using Pearson chi-square test with normal approximation. Detailed sample size calculation steps, including the statistical formula, parameter justification, and sensitivity analysis results, are provided in the Supplementary Material.

### 2.8 Management of loss to follow-up (LTFU)

To mitigate LTFU risks during the 36-month follow-up, high-risk participants (e.g., those with unstable residence or long distance from study sites) are required to provide alternative contacts (Li et al., 2023), while dedicated coordinators use a tiered reminder system (phone, SMS/WeChat) to notify participants of scheduled follow-up visits; within 48 h of a missed visit, additional contact attempts are made via primary and alternative channels, and a 10% LTFU allowance has been incorporated into the sample size calculation (150 participants per arm) to ensure 80% statistical power for the primary outcomes (Ren et al., 2016; Li et al., 2023).

### 2.9 Statistical analysis

The statistical analyses will be carried out in SAS v9.4 by independent statisticians. Descriptive statistical analysis will be performed by calculating the mean ± SD, median (minimum-maximum, or lower quartile-upper quartile) for continuous variables and counts (percentages) for categorical variables. The statisticians will perform chi-square test or Fisher’s exact test for categorical variables and Student’s test or Wilcoxon rank sum test for continuous variables. It will be considered statistically significant if p value <0.05. The efficacy analysis will use intention-to-treat, per-protocol, and as-treated populations. The concentration of anti-TB drugs will be determined using a validated LC-MS method at the Center for Pharmaceutical Analysis and Testing, School of Pharmaceutical Sciences, Shandong University. NONMEM 7.4 will be used to determine whether the PD targets have been achieved for each participant. Detailed statistical analyses are provided in the Supplementary Material.

### 2.10 Safety and AEs monitoring

All AEs will be precisely monitored and recorded from the moment of obtaining informed consent to the follow-up visit. Serious AEs will be reported according to the local regulations and procedures.

The safety monitoring committee of this trial consists of Professor Johannes van den Anker from the Shandong University and Professor Evelyne Jacqz-Aigrain from Paris Cité University. And the data monitoring will be performed by Institute of Clinical Pharmacology, Shandong University. The monitoring will be in accordance with the requirements of the GCP guidelines.

## 3 Discussion

Spinal tuberculosis is a bone infection caused by *Mycobacterium tuberculosis* (Mtb), located in deep tissues, and its treatment currently faces multiple challenges. From a pharmacological perspective, bone tissue is often poorly vascularized, resulting in inadequate drug penetration. For example, the bone concentration of rifampin is only 20%–40% of its plasma concentration (Thabit et al., 2019). From a bacterial perspective, the formation of Mtb biofilms has been confirmed *in vitro*, in animal infection models (in the bone) and in patients (in the lungs) (Trivedi et al., 2016; Chakraborty et al., 2021; Staats et al., 2021). Biofilm formation contributes to the development of drug resistance. Furthermore, Mtb can evade immune responses by inhibiting macrophage maturation, lysosomal acidification, and suppressing oxidative stress, apoptosis, and autophagy, allowing the bacteria to remain dormant within the host (Zhai et al., 2019). This persistence of bacteria makes clinical relapse more likely. Therefore, the bacteria responsible for spinal tuberculosis are particularly difficult to fully eradicate, which is why treatment often requires a prolonged duration (12–18 months). However, the contradiction arises in that the toxicity of the drugs and the patients’ adherence issues, etc. During such long treatments may lead to interruptions, undermining the very success of the therapy. This is the core dilemma in treating spinal tuberculosis.

Therefore, shortening the treatment duration has become a key strategy, with related studies already conducted on non-tuberculous osteoarticular infections (such as prosthetic joint infections and osteomyelitis) (Yen et al., 2019; Benavent et al., 2021; Scheper et al., 2022). For pulmonary tuberculosis, a 4-month treatment regimen also has been proven to be non-inferior to a 6-month regimen (Dorman et al., 2021). Shortening the treatment duration for spinal tuberculosis may be achievable, and the key lies in selecting the right drugs that can address the challenges in treating spinal tuberculosis. The anti-tuberculosis efficacy of rifapentine and moxifloxacin has been confirmed in a 4-month regimen for pulmonary tuberculosis (Dorman et al., 2021). Moreover, animal data on rifapentine show that, at the same dosing levels as rifampin, rifapentine achieves higher concentrations than rifampin in bone, except in the medulla (Iversen et al., 1983). Additionally in an osteoblast infection model, the minimum biofilm eradication concentration of rifapentine for *S. aureus* isolates is lower than that of rifampin (Abad et al., 2020). Moxifloxacin also exhibits good penetration into bone and joints (Malincarne et al., 2006). Rifapentine demonstrates significantly lower hepatotoxicity compared to rifampin (Dorman et al., 2021) and moxifloxacin demonstrates comparable tolerability to ethambutol, a medication widely regarded as having a high safety profile (Conde et al., 2009). Therefore, we hope to shorten the treatment duration to 6 months by replacing the drugs in the anti-tuberculosis regimen with rifapentine and moxifloxacin.

Finally, conducting this study in China is highly feasible and of great significance, as China ranks third among the 30 countries with the highest TB burden in WHO data (WHO, 2024). Among the various forms of extrapulmonary tuberculosis, spinal tuberculosis is the most prevalent, constituting roughly 1%–3% of all tuberculosis cases (Nene et al., 2019; WHO, 2022). Moreover, tuberculosis is a key disease for prevention and control in China, and its treatment is already covered by medical insurance (National Disease Control and Prevention Administration, 2023; National Disease Control and Prevention Administration, 2024; National Healthcare Security Administration, 2024).

Therefore, this study is feasible and profoundly significance. The outcomes of this study have the potential to significantly influence clinical practice by providing robust evidence on the viability of a shorter, more convenient treatment option for spinal tuberculosis.
