# Supplementary material for: Rifapentine- and moxifloxacin-containing short-course regimens for mild spinal tuberculosis: study protocol for a multicenter, randomized, non-inferiority phase II clinical trial
Source: Front Pharmacol. 2025 Dec 19;16:1684771. doi: 10.3389/fphar.2025.1684771 (PMC12757414; doi:10.3389/fphar.2025.1684771)
Supplement: Supplementary file 1 [file DataSheet1.docx]

Supplementary Material

# Sample Size

To ensure the rigor of the study’s analytical framework, the sample size was determined based on the study’s primary efficacy outcome (TB-recurrence rate at 24 months post-treatment) and a non-inferiority design. All parameters were justified using clinical evidence and statistical principles.

# Core Assumptions and Justification

- Baseline recurrence rate: A 5% recurrence rate was assumed for the empirical long-course regimen (2RHZE/10RH) group. This assumption was derived from prior clinical data a pilot study on spinal TB (Nene et al., 2019) reported a 4.7% recurrence rate in the 12-month treatment arm while a large multi-center study (Ren et al., 2016) demonstrated a 5.2% recurrence rate for drug-sensitive spinal TB treated with standard long-course regimens. The rate was rounded to 5% to account for minor variability across populations.
- Non-inferiority margin (Δ): A one-sided non-inferiority margin of 6.6% was pre-specified. This margin was justified by two key considerations:
  - Clinical relevance: For mild spinal TB, a recurrence rate difference ≤ 6.6% is clinically acceptable as the short-course regimen offers substantial benefits (reduced toxicity, improved adherence) that outweigh a small, non-clinically meaningful increase in recurrence (WHO, 2022);
  - Regulatory alignment The margin is consistent with WHO guidelines for non-inferiority TB trials (Δ ≤ 10% for recurrence outcomes) and design standards of similar osteoarticular TB studies (Dorman et al., 2021).
- Statistical power and type I error: Statistical power was set at 80% (1−β = 0.8) with a one-sided type I error rate (α) of 0.05. This is a standard configuration for phase II clinical trials balancing the need to detect meaningful treatment differences with feasibility (e.g., avoiding excessively large sample sizes that would prolong participant recruitment).
- Dropout rate: A 10% dropout/LTFU rate was incorporated based on data from Chinese multi-center spinal TB trials with ≥24 months of follow-up (Ren et al., 2016; Li et al., 2023)—in these studies, LTFU rates were consistently <8%. The 10% buffer ensures sufficient statistical power even if LTFU exceeds expected levels.

# Core Assumptions and Justification

The sample size was calculated using the formula for non-inferiority tests of binary outcomes (Pearson chi-square test with normal approximation):

$$n=\frac{{(Z_{1-\alpha}+Z_{1-\beta})}^{2}\times[p_{0}(1-p_{0})+p_{1}(1-p_{1})]}{\Delta^{2}}$$

Where:

- $p_{0}$ = 0.05 (recurrence rate in long-course group), $p_{1}$= 0.05 (assumed recurrence rate in short-course group, non-inferiority hypothesis),

- $Z_{1-\alpha}$ = 1.645 (one-sided α=0.05), $Z_{1-\beta}$ = 0.842 (power=80%),

- $\Delta$ = 0.066 (non-inferiority margin).

The calculation yielded 136 participants per treatment arm. Accounting for the 10% LTFU rate, the sample size was rounded up to 150 participants per arm (total n = 300) ensuring 80% power to detect the pre-specified non-inferiority margin. This calculation was verified using SAS v9.4.

# Sensitivity Analysis

A sensitivity analysis was conducted to assess the impact of deviations from core assumptions

- If the recurrence rate in the long-course group increases to 7% (worst-case scenario), the sample size of 150 participants per arm still maintains 78% statistical power (closely approaching the 80% target);
- If LTFU rises to 15%, power remains ≥75%—a level deemed acceptable for phase II trials per International Council for Harmonisation of Technical Requirements for Pharmaceuticals for Human Use (ICH) E8 guidelines (ICH, 2021).

# Statistical Analysis

All analyses will be conducted in SAS v9.4 by independent statisticians with pre-specified study populations and hypotheses.

# Pre-Specified Study Populations

- Intention-to-Treat (ITT) Population: All randomized participants. Serves as the primary population for efficacy analysis, preserving randomization benefits and reflecting real-world practice (ICH E9 guidelines (ICH, 1998)).
- Per-Protocol (PP): Participants who completed full treatment, adhered to the protocol (missing <20% of doses), and had no major protocol violations. Used for sensitivity analysis to assess non-adherence impact.
- As-Treated (AT): Participants analyzed by the actual regimen received. Used for safety analysis to avoid confounding from treatment switching.

# Efficacy Analysis

- Binary outcomes (recurrence rate, clinical cure): Pearson chi-square test (ITT population) for group comparisons; Fisher’s exact test if expected cell counts <5. Non-inferiority of the short-course regimen is confirmed if the lower bound of the one-sided 95% CI for recurrence rate difference (short-course − long-course) ≥−6.6%.
- Continuous outcomes (ESR, PK parameters): Independent samples t-tests (unadjusted) and ANCOVA (adjusted for baseline, primary approach) for normally distributed data; Wilcoxon rank-sum tests (unadjusted) and quantile regression (adjusted for baseline) for non-normal data. Baseline adjustment mitigates open-label bias and improves estimate precision.
- PK/PD analyses: NONMEM software estimates PK parameters (AUC_0-24h_, C_max_). PD target attainment rate (rifapentine AUC_0-24h_/MIC, moxifloxacin AUC_0-24h_/MIC) is compared between groups via Pearson chi-square test (Asin-Prieto et al., 2015).

# Safety Analysis

- AEs: Fisher’s exact test (AT population) compares the proportion of grade 3+ AEs; Pearson chi-square test analyzes specific AE incidence (e.g., hepatotoxicity). 95% CIs are reported for group differences.

# Statistical Significance and Reporting

All tests will be two-sided except for the primary non-inferiority analysis (one-sided). A p-value <0.05 will be considered statistically significant for secondary outcomes and safety analyses. All results will be reported with 95% CIs (one-sided for the primary non-inferiority analysis, two-sided for all other analyses) in compliance with CONSORT guidelines for randomized controlled trials including transparent reporting of the open-label design and measures to mitigate associated bias.

**Reference**

Asin-Prieto, E., Rodriguez-Gascon, A., and Isla, A. (2015). Applications of the pharmacokinetic/pharmacodynamic (PK/PD) analysis of antimicrobial agents. J Infect Chemother 21(5), 319-329. doi: 10.1016/j.jiac.2015.02.001.

Dorman, S.E., Nahid, P., Kurbatova, E.V., Phillips, P.P.J., Bryant, K., Dooley, K.E., et al. (2021). Four-Month Rifapentine Regimens with or without Moxifloxacin for Tuberculosis. N Engl J Med 384(18), 1705-1718. doi: 10.1056/NEJMoa2033400.

ICH (1998). "Statistical Principles for Clinical Trials (ICH E9)".).

ICH (2021). "E8(R1) General considerations for clinical trials".).

Institute, N.C. (2017). "Common Terminology Criteria for Adverse Events (CTCAE) Version 5.0".).

Li, T., Yan, X., Du, X., Huang, F., Wang, N., Ni, N., et al. (2023). Extrapulmonary tuberculosis in China: a national survey. Int J Infect Dis 128, 69-77. doi: 10.1016/j.ijid.2022.12.005.

Nene, A.M., Patil, S., Kathare, A.P., Nagad, P., Nene, A., and Kapadia, F. (2019). Six versus 12 Months of Anti Tubercular Therapy in Patients With Biopsy Proven Spinal Tuberculosis: A Single Center, Open Labeled, Prospective Randomized Clinical Trial-A Pilot study. Spine (Phila Pa 1976) 44(1), E1-E6. doi: 10.1097/BRS.0000000000002811.

Ren, H.L., Jiang, J.M., Wang, J.X., Qu, D.B., and Chen, J.T. (2016). Is duration of preoperative anti-tuberculosis treatment a risk factor for postoperative relapse or non-healing of spinal tuberculosis? Eur Spine J 25(12), 3875-3883. doi: 10.1007/s00586-016-4496-2.

WHO (2022). "WHO consolidated guidelines on tuberculosis. Module 4: treatment - drug-resistant tuberculosis treatment, 2022 update".).
